# Bladder cancer biomarkers

**DOI:** 10.37349/etat.2025.1002301

**Published:** 2025-03-25

**Authors:** Dominik Godlewski, Dorota Bartusik-Aebisher, Sara Czech, Jakub Szpara, David Aebisher

**Affiliations:** University of Bari, Italy; IRCCS Istituto Romagnolo per lo Studio dei Tumori (IRST) “Dino Amadori”, Italy; ^1^Medical Center In Łańcut, 37-100 Łańcut, Poland; ^2^Department of Biochemistry and General Chemistry, Medical College, The Rzeszów University, 35-959 Rzeszów, Poland; ^3^English Division Science Club, Medical College, The Rzeszów University, 35-959 Rzeszów, Poland; ^4^Department of Photomedicine and Physical Chemistry, Medical College, The Rzeszów University, 35-959 Rzeszów, Poland

**Keywords:** Bladder cancer, biomarkers, diagnostic, prognostic, predictive biomarkers, monitoring, urothelial cancer

## Abstract

Bladder cancer (BCa) is among the most frequently diagnosed urinary tract cancers, characterized by a high recurrence rate and significant clinical heterogeneity. Effective diagnosis and treatment of BCa demand continuous advancements in medical technologies, particularly given the limitations of classical methods such as cystoscopy and urine cytology. A comprehensive search of PubMed and Web of Science was conducted using relevant keywords to structure this narrative review. Additionally, specialist journals were reviewed. Only articles in English were included, with selection based on titles, abstracts, and availability of full texts. In recent years, biomarkers have emerged as crucial tools complementing traditional techniques, providing more precise, sensitive, and non-invasive methods for early detection, prognosis, and monitoring treatment response in BCa. Molecular, genetic, and protein biomarkers enable a deeper understanding of BCa biology, creating opportunities for personalized therapy tailored to individual patient needs. However, despite their potential, certain challenges remain, including standardization, validation, and integration into routine clinical practice. This review highlights recent advancements in BCa biomarkers and their transformative potential in oncological care. It underscores the importance of incorporating these innovations to refine diagnostic and therapeutic approaches, ultimately improving patient outcomes. Modern diagnostic and prognostic tools for BCa can enhance treatment outcomes by enabling early disease detection and reducing recurrence risks. This progress promises to improve patients’ quality of life by minimizing disease burden and fostering effective, tailored care strategies.

## Introduction

Bladder cancer (BCa) is one of the most commonly diagnosed malignancies of the urinary system, developing from epithelial cells lining the bladder wall, which plays a key role in storing urine. In developed countries, urothelial cancer is the predominant form of BCa, accounting for more than 90% of all cases [1]. In 2022, an estimated 614,098 new cases of BCa were reported worldwide, with a total of 199,922 deaths. The age-standardized incidence rate (ASIR) shows considerable geographical variation, but projections indicate an increase in the number of cases in the coming years, which may increase the burden on global healthcare systems [2].

### Epidemiology and significance of the problem

**Figure 1:**
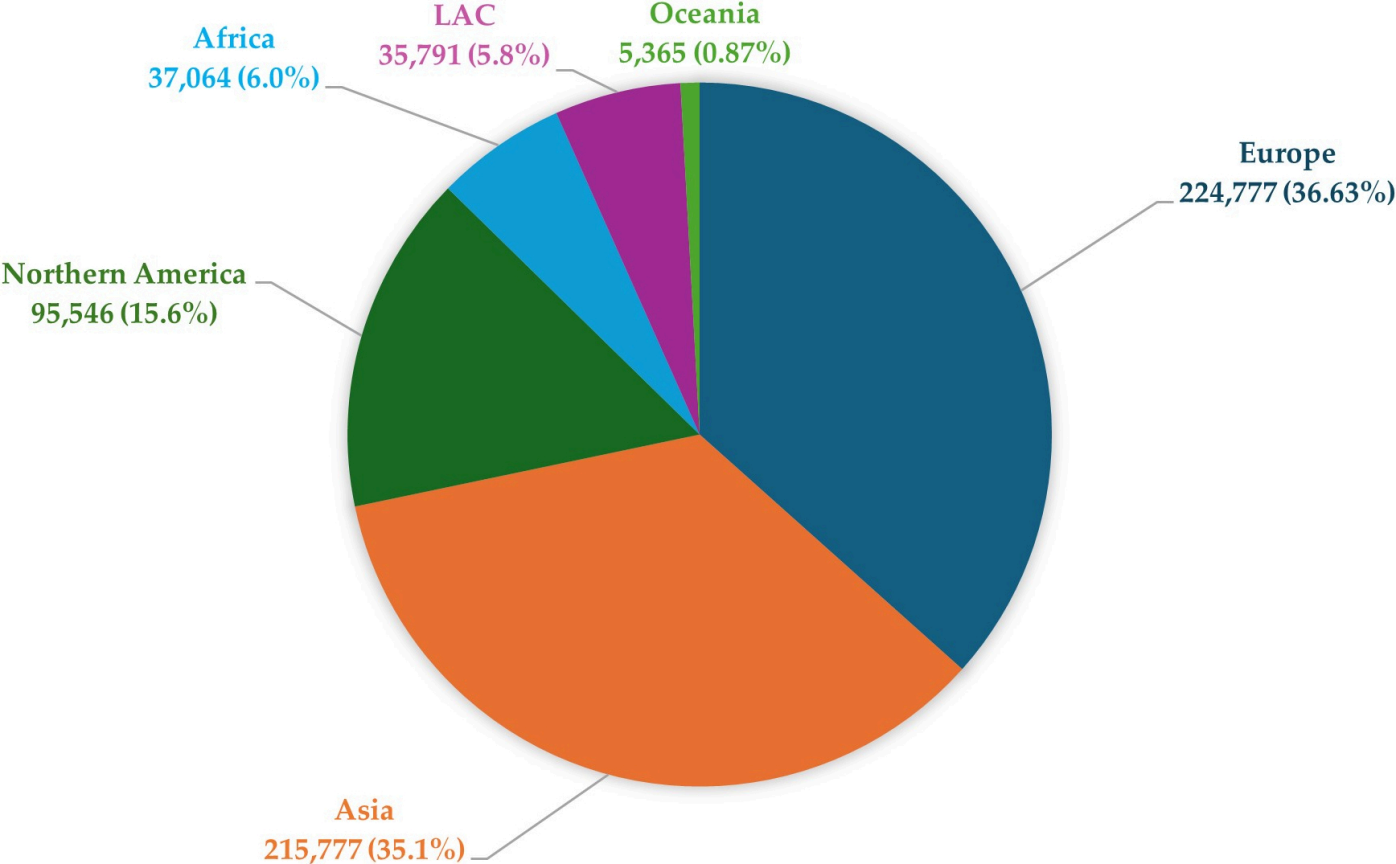
Graph shows the global distribution of new cases of bladder cancer in 2022

BCa shows significant variation in incidence by geographic region, as confirmed by data from the World Health Organization (WHO). Smoking incidence risks (SIRs) are almost three times higher in developed countries than in less affluent regions, at 9.5 cases per 100,000 inhabitants in developed countries and 3.3 cases per 100,000 inhabitants in less developed countries [3]. These differences may be related to various risk factors, such as smoking, obesity, alcohol consumption, and a diet rich in red meat, which are more common in higher-income countries. Epidemiological analyses indicate that the incidence of BCa is increasing in high-income countries, while mortality related to this cancer is decreasing [4]. This phenomenon may be due to advances in diagnostics, early detection, and more effective treatment of BCa, available in more advanced health care systems. The level of socioeconomic development may significantly impact the risk of developing this cancer [5]. The global distribution of new cases of BCa in 2022 is presented in Figure 1. Europe and Asia account for the highest proportions, followed by Northern America, while other regions represent smaller shares.

### Risk factors for BCa development

Risk factors for BCa incidence is greater in men, BCa tends to be more aggressive in women (greater muscle invasion rate), suggesting that oestrogen inhibits tumour initiation but promotes invasion. Differential expression of oestrogen receptor 1 (ESR1; also known as ERα) and ESR2 (also known as ERβ) in human BCa s implicates different roles in bladder tumorigenesis [6, 7].

**Figure 2:**
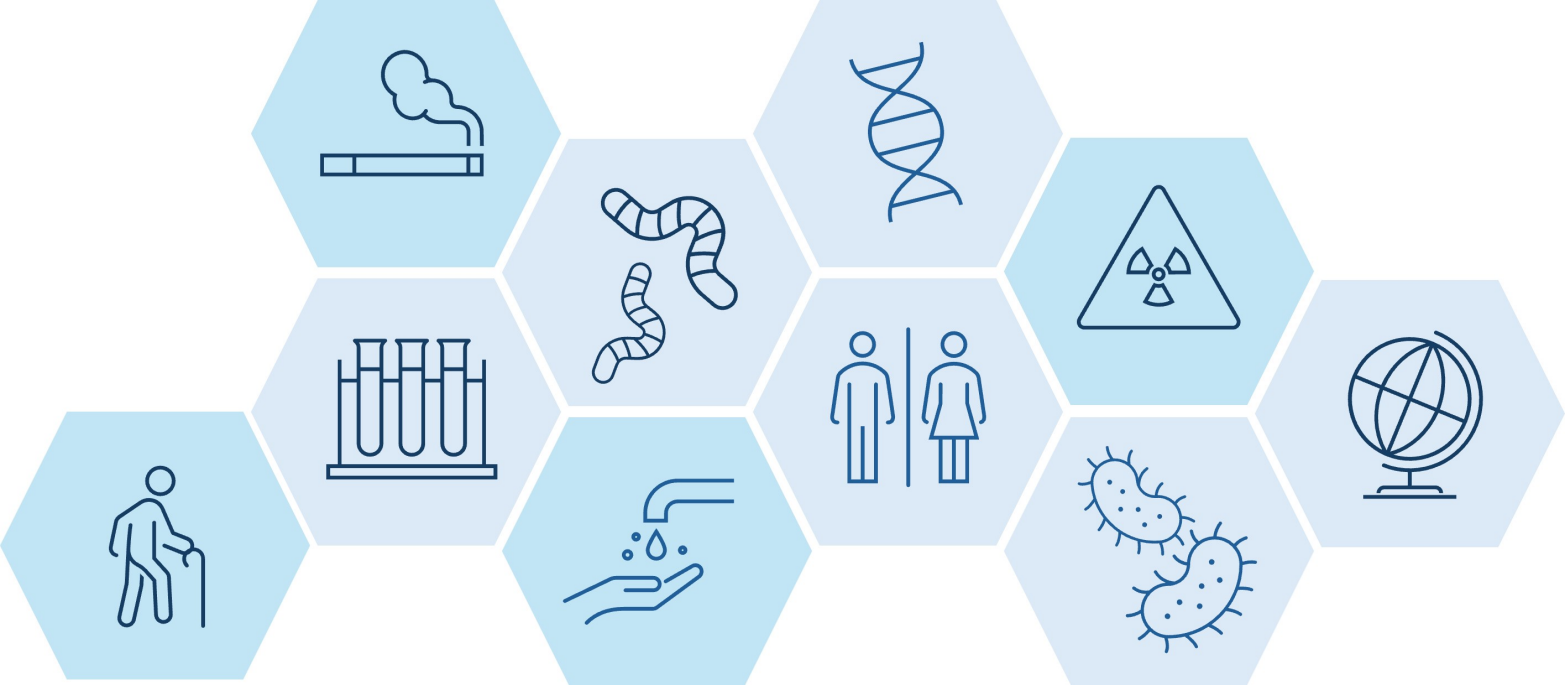
Illustration shows key risk factors for developing bladder cancer

The development of BCa is associated with various risk factors, the most important of which are tobacco smoking, exposure to chemicals and chronic inflammation of the bladder [1, 8]. Differences in the risk of disease between men and women are explained not only by smoking habits, but also by the influence of hormones. Androgens, typical for men, promote the development of BCa, while estrogens may play a protective role, which may explain the lower incidence of the disease in women [9]. Below is Figure 2, which presents the main risk factors associated with the development of BCa. The main factors include smoking, exposure to chemical carcinogens (such as industrial dyes and arsenic compounds), parasitic infections (such as schistosomiasis), aging, previous chemotherapy or radiotherapy, chronic bladder infections and irritations, genetic factors, gender (higher risk in men), race (higher risk in whites), and exposure to arsenic in drinking water.

### Symptoms

BCa itself isn’t usually painful in the early stages. Blood in the urine (hematuria) is often the first noticeable symptom. In advanced stages, however, pain may occur in the lower back, pelvis, or during urination. BCa most often manifests itself with hematuria, which may be visible to the naked eye (macroscopic hematuria) or detected only during microscopic examination (microscopic hematuria) [10].

Additional symptoms may include frequent urination and unexplained pelvic pain. Early diagnosis and treatment are crucial, as BCa can occur in both a non-invasive form, limited to the bladder mucosa, and in an invasive form, penetrating the deeper layers of the bladder wall and potentially leading to metastases. At the time of diagnosis, approximately 75% of cases are non-muscle invasive BCa (NMIBCa), while 25–30% of cases already show an advanced, invasive form of the disease, muscle-invasive BCa (MIBCa) [1, 2].

### Histological types of BCa

Table 1 presents the main histological types of BCa, their incidence, and clinical and pathological characteristics. Each type of cancer differs in both biology and etiology, which has a significant impact on the course of the disease, prognosis, and available therapeutic options. Histological types of BCa include the most common urothelial tumors as well as rarer but often more aggressive forms such as squamous cell carcinoma, adenocarcinoma, and sarcoma [1].

**Table 1:** Main histological types of BCa, incidence, characteristics

**Histological type**	**Incidence**	**Characteristics**
Urothelial cancer	90%	The most common type, develops from the transitional epithelial cells lining the urinary bladder. It comes in non-invasive (NMIBCa) and invasive (MIBCa) forms. It is the predominant type in developed countries.
Squamous cell carcinoma	3–5%	Associated with chronic inflammation, most often caused by schistosome infection (especially in endemic regions) or chronic cystitis. It is characterized by an aggressive course.
Adenocarcinoma	< 2%	It arises from glandular cells; it may occur primarily in the bladder or be secondary (metastatic) from other locations. It is often associated with congenital defects such as persistent urachus.
Small cell carcinoma	< 1%	A very rare, highly aggressive type of cancer with a rapid progression, often with metastases at diagnosis. Histologically similar to small cell lung cancer.
Sarcoma	< 1%	Rare; originates from muscle tissue or other mesenchymal structures of the bladder. More common in children and young adults, known as rhabdomyosarcoma.
Mixed cancer	Variable	May contain features of different types, e.g., urothelial and squamous. Mixed cancer shows a more aggressive course compared to pure urothelial tumors.

BCa: bladder cancer; MIBCa: muscle-invasive BCa; NMIBCa: non-muscle invasive BCa

### The importance of early diagnosis and disease monitoring

Early diagnosis of BCa plays a significant role in reducing mortality and improving patient outcomes, especially in the context of low-grade malignancies such as NMIBCa. Early detection and treatment of the disease allows for the use of less aggressive methods, which increases the effectiveness of therapy and reduces the risk of progression to more advanced stages such as MIBCa. From a public health perspective, early detection of the disease can significantly reduce healthcare costs, as later stages of the disease require more expensive medical care and have a poorer prognosis [11]. The standard diagnostic methods used to detect BCa are cystoscopy and urine cytology. Cystoscopy allows direct visualization of the bladder and assessment of neoplastic lesions [12].

However, this method is highly invasive, expensive, and may be uncomfortable for the patient. Furthermore, cystoscopy can be ineffective in detecting very small lesions, which may lead to missing early stages of BCa. Urine cytology, although less invasive and useful in detecting high-grade malignancies, has limited sensitivity in the case of low-grade lesions, which affects its value in the early diagnosis of BCa [13]. Due to the limitations of conventional methods, more and more attention is being paid to non-invasive biomarker tests that could complement current diagnostic procedures. These tests, based on the detection of circulating cell-free DNA (cfDNA) or specific genetic mutations in urine, show promising results as a complementary tool for early detection of BCa [14].

These biomarkers are particularly valuable because they allow monitoring of disease recurrence without the need for frequent and expensive cystoscopies [15]. Thus, the analysis of the potential of biomarkers opens perspectives for future research and clinical implementations that could improve the quality of life of patients and treatment outcomes. The following chapters describe the most important biomarkers of BCa, which may play a key role in the future in the context of diagnosis, monitoring and personalization of treatment of this disease [16].

## Types of biomarkers in BCa

### Definition and main division of biomarkers

There are many reports in the medical literature on biomarkers, including those in the context of BCa. These biomarkers can be divided into three main categories: diagnostic, prognostic, and predictive. A biomarker, as broadly defined, is a characteristic that is objectively measured and evaluated as an indicator of normal biological processes, disease processes, or response to therapeutic interventions [17].

#### Diagnostic biomarkers

Diagnostic biomarkers in BCa play an important role in disease detection, especially in the context of patients with symptoms of hematuria. While macroscopic hematuria is associated with an approximately 20% risk of cancer, microscopic hematuria carries a much lower risk of only 0.43%, although it is common in the adult population. In this context, biomarkers can serve as a tool for selecting patients requiring further diagnostics. The use of genetic panels obtained from urine samples has become a promising method for detecting BCa. An example is the CxBladder test, which measures the expression of five mRNAs associated with BCa and achieves a sensitivity of 82% and a specificity of 85%. Moreover, there are many other tests, analyzing, among others, mutations in the fibroblast growth factor receptor 3 (*FGFR3*), *TERT*, *HRAS* genes [18]. Commonly studied diagnostic biomarkers such as nuclear matrix protein 22 (NMP22), UroVysion, and uCyt+ have shown varying sensitivity and specificity, which may be due to differences in study populations and study methodology. UroVysion has the best diagnostic performance (AUC 0.876), and NMP22 is often used in combination with cytology. Despite promising results, further validation of these biomarkers is needed to confirm their usefulness in routine diagnostics [19].

#### Prognostic biomarkers

A prognostic biomarker is a biological indicator that allows predicting the course of the disease, regardless of the treatment used. It assesses the probability of disease recurrence or patient survival, providing valuable information on the risk associated with disease progression. OLFML2B has been identified as a new, robust prognostic biomarker in BCa. In multiple independent cohorts, high expression of this gene has been shown to be associated with poorer patient prognosis. OLFML2B expression increases with increasing clinical stage of the cancer, suggesting that this gene promotes tumor progression. OLFML2B is also strongly correlated with tumor-associated macrophage infiltration, suggesting its role in shaping the tumor microenvironment. Additionally, studies indicate that downregulation of OLFML2B leads to inhibition of BCa cell migration and proliferation, making it a potential therapeutic target [20]. A 2024 study found that CD276 is highly overexpressed in BCa tissues compared with adjacent healthy tissues. CD276 expression was significantly higher in patients with more advanced disease, including lymph node or distant metastases, and in patients with high-grade disease. High CD276 expression was also associated with shorter overall survival (OS) and progression-free survival (PFS), suggesting its potential as a prognostic biomarker. Gene set enrichment analyses (GSEA) revealed that CD276 is associated with extracellular matrix (ECM) pathways, which may indicate its role in promoting tumor progression by influencing tumor cell migration and invasion. These results suggest that CD276 may be a useful diagnostic tool and therapeutic target in BCa [21].

#### Predictive biomarkers

Predictive biomarkers play a key role in oncology, as they allow for prediction of patient response to specific therapies. In the case of BCa, biomarkers can help identify patients who will benefit from specific treatments, such as neoadjuvant chemotherapy or immunotherapy, minimizing unnecessary therapies with high toxicity. Examples of such biomarkers include mutations in the FGFR or expression of the PD-L1 ligand, which indicate the efficacy of targeted therapies and thus increase the chances of better treatment outcomes [22]. A 2024 article showed that biomarkers related to molecular subtypes (e.g., basal types), EMT (epithelial-mesenchymal transition), and immune cells (CD8+ T cells) can predict response to neoadjuvant chemotherapy. However, tumor heterogeneity poses a challenge to the accuracy of these markers. Further studies are needed to confirm their utility in routine clinical practice and to improve the efficacy of BCa treatment [23]. In recent years, studies on molecular subtypes of BCa (MIBCa) and predictive biomarkers in the treatment of this tumor have shown great potential in predicting the response to therapy, as described in a 2022 article. For example, molecular subtypes such as “luminal” and “basal”, which can be identified by the biomarkers KRT5/6 and GATA3, can help predict the efficacy of neoadjuvant chemotherapy. Studies have shown that patients with the basal subtype are more likely to respond positively to chemotherapy, while the luminal subtype is associated with a better long-term prognosis. Furthermore, studies on other markers such as PDGFRB and collagen proteins indicate that they may be useful in further predicting the response to therapy in patients with MIBCa [24].

### Molecular biomarkers

#### Genetic

Mutations in the *FGFR3* and *TP53* genes are considered to be extremely important biomarkers in BCa, enabling much more precise classification and prediction of the course of the disease. Mutations in the *FGFR3* gene, which cause constitutive activation of this FGFR, occur mainly in superficial (pTa) tumors of a lower malignancy grade. Such mutations are detected in about 65% of superficial pTa tumors and less frequently in tumors of more advanced grades [25, 26]. *FGFR3* mutations are often associated with a better prognosis and show greater susceptibility to targeted therapies, such as FGFR inhibitors, and therefore are a promising prognostic and predictive biomarker in BCa [27]. In turn, mutations in the *TP53* gene, which is a key tumor suppressor gene, occur more often in more aggressive, invasive bladder tumors. These mutations are associated with high-grade malignancy and play a role in BCa progression through disruption of cell cycle regulation and apoptosis. Carcinoma in situ (CIS) and muscle-invasive tumors (pT2–pT4) exhibit high rates of *TP53* mutations, suggesting that this biomarker is an indicator of disease progression and may be a valuable predictor of more aggressive forms of BCa [25, 26].

*FGFR3* and *TP53* mutations are often mutually exclusive, suggesting that they participate in distinct molecular pathways of BCa. Early superficial tumors are more likely to have *FGFR3* mutations, whereas invasive forms of cancer are more likely to have *TP53* mutations. This distinction could be used to create molecular subtypes of the tumor, which could facilitate the development of more targeted treatments based on specific genetic profiles [25–27]. Another gene that may play a key role in the development of BCa is the *HRAS* gene—especially through the Gly12Val mutation, which leads to protein hyperactivity and uncontrolled division of cancer cells. According to a 2015 study, HRAS mutations increase the risk of disease recurrence after treatment, making it an important biomarker in predicting the course of BCa [28]. In turn, an article published in 2018 says that RAS inhibitors, such as salirasib, can inhibit the growth of cancer cells, suggesting their potential therapeutic value, although they require high concentrations for their effectiveness [29]. The *RB1* gene is an important biomarker in BCa, especially in the context of response to immune therapies. Studies have shown that *RB1* mutations in combination with *TP53* mutations correlate with a higher rate of genomic mutations, increased mutational burden, and a stronger presence of immune effectors such as cytotoxic lymphocytes and NK cells. These co-occurring mutations support the response to immune checkpoint inhibitors (ICIs), making RB1 a valuable predictor for therapy, especially in advanced cases of BCa [30, 31]. Furthermore, RB1 has been identified as a differentially mutated driving gene in both smokers and nonsmokers, and the rate of *RB1* mutations increased by as much as twofold (*P* = 0.008) in subjects who regularly smoked tobacco products. It has also been emphasized that *RB1* mutations associated with smoking may cause bladder carcinogenesis by inhibiting the cytochrome P450 pathway and regulating the tumor’s immune microenvironment [32]. Gene amplification and deletion play an important role in the diagnosis and monitoring of BCa. A study published in 2020 shows that these changes can support better prognosis and prediction of treatment response. Detection of deletions in chromosomes such as 9p21 and 17p is particularly important because they are associated with a more aggressive course of the disease and a higher risk of relapse. Genetic changes in the bladder, such as deletion of the 9p21 region, affect the functioning of key proteins, including the CDKN2A protein, which promotes the development of low-specificity tumors and increases the risk of relapse [33].

#### Epigenetic

In BCa, epigenetic changes play a key role, including in DNA, through hypermethylation of tumor suppressor genes such as *CDKN2A* and *HOXA9*, which is associated with aggressive disease progression. Hypermethylation of these genes can be detected in patients’ urine, which allows for their monitoring without the use of invasive methods, which is certainly a very big advantage. Epigenetic urine tests, such as UroMark, can potentially improve the diagnosis of BCa and predict the response to treatment, supporting clinical therapeutic decisions [34]. DNA methylation, consisting of the attachment of a methyl group to cytosine in CpG islands, is an extremely important biomarker in the diagnosis and treatment of BCa. In this cancer, hypermethylation of tumor suppressor gene promoters leads to their silencing, which promotes disease progression, cell invasion and metastasis. Studies indicate that CpG island methylation may vary with the stage and aggressiveness of cancer, making it a prognostic tool and a potential indicator of response to therapy. Profiling methylation in patients’ urine sediment shows consistency with tumor patterns, which can help in diagnosis and monitoring treatment response. Additionally, reduced global methylation in repetitive regions of the genome in BCa promotes chromosomal instability, which can stimulate mutations and tumor development [35].

A 2023 article discusses the role of RASSF1 methylation—one of the genes that is relatively frequently methylated—as a potential biomarker of BCa, especially in patients with neurogenic lower urinary tract dysfunction (NLUTD). RASSF1 methylation was analyzed in urine samples, which showed a higher percentage in patients with NLUTD compared to the control group. The results suggest that RASSF1 methylation may be useful in noninvasive monitoring of patients with NLUTD who are at increased risk. However, it is worth adding here that further studies are required on the possibility of using this method in clinical use [36].

Moreover, differential methylation of genes such as *CDH1* has been observed in BCa samples. In cases associated with *Schistosoma* infection, a higher number of differentially methylated genes, including *CDH1*, was noted compared to non-*Schistosoma*-related cases. The methylation of CDH1 may has a potential as a prognostic biomarker, warranting further investigation in the context of BCa [36, 37].

hOGG1, as a biomarker, plays an important role in detecting the risk of cancer, including BCa. It is an enzyme responsible for DNA repair, removing 8-oxoguanine, damage caused by reactive oxygen species (ROS) [37]. Variants such as Ser326Cys and Ser326Ser were associated with a higher risk of BCa recurrence and were considered potential prognostic indicators, especially in NMIBCa [38].

#### MicroRNA (miRNA)

MicroRNAs (miRNAs) are short, non-coding RNA molecules that regulate gene expression and play a key role in cellular processes related to BCa. Due to their stability and presence in urine, miRNAs offer potential as biomarkers. Meta-analyses have shown that multi-miRNA assays, especially in urine sediment, achieve higher sensitivity and specificity (80.9% and 83.1%, respectively) compared to single assays, which significantly increases their potential clinical utility. The variability of miRNA sensitivity and specificity makes them adaptable to various diagnostic purposes, thus achieving advantages over traditional tumor markers, while offering new perspectives in the development of personalized tools for early detection of BCa [39]. Moreover, in BCa, changes in miRNA expression are important for cell cycle, apoptosis, metastasis, drug resistance, and other key tumor functions [39]. In BCa, there is a decreased expression of some miRNAs (such as miR-145, miR-133a), which may act as tumor suppressors, and an increased expression of other miRNAs (such as miR-21 and miR-200b), which act as oncogenes. Deregulation of miRNAs is also associated with epigenetic modifications, which may contribute to the progression of BCa itself. miRNAs may also differ depending on the tumor type, its stage, or patient prognosis. Below is a table with selected miRNAs associated with BCa [40, 41] (Table 2).

**Table 2:** Selected miRNAs in bladder cancer

**miRNAs**	**Biological role**	**Clinical significance**	**Reference**
miR-21	Apoptosis and participation in the mesenchymal transition	Diagnosis of the disease and prognosis for its development	[40]
miR-96 miR-183	Supporting cell growth, participating in their migration and apoptosis	Diagnosis of the disease and prognosis for its development	[42]
miR-29a-3p	Playing an extremely important role in maintaining a proper methylation profile	Stratification into high and low risk groups—its presence indicates a good prognosis	[43]
miR-124	Participation in both migration and invasion processes	Diagnosis of the disease and prediction of the response to a given therapy	[44]
miR-940	It may affect the proliferation, migration, and invasion of cells	Regulation of signaling pathways, such as Wnt/β-catenin, and target genes, such as *INPP4A* and *GSK3β*	[45]
miR-23b	Extremely important in the transition between epithelium and mesenchyme	Therapeutic purpose	[46]
miR-205	Participation in apoptosis processes and the cell cycle, important in invasion processes, very important role in the transition between epithelium and mesenchyme	It is a therapeutic target, but it also plays a significant role in the prognosis of the disease	[47]
miR-214	Very important in inhibiting the processes of migration, invasion and proliferation	Diagnosis of the disease and prognosis for its development	[48]
miR-590-3p	It plays an important role in the process of carcinogenesis	It is an important goal in therapy	[49]
miR-126	It plays a key role in the regulation of blood vessels and angiogenesis, influencing endothelial cell migration and adhesion. It also acts as a tumor suppressor, inhibiting cancer cell proliferation and invasion	It is a promising biomarker of bladder cancer, enabling detection of the disease in urine samples. Due to its high specificity and sensitivity, it can be used in non-invasive diagnostic tests	[40]
miR-141-3p	It acts as a regulator of oncogenic pathways, controlling processes related to cell proliferation, apoptosis and migration	It is a promising biomarker for cancer diagnosis and for distinguishing malignant from benign lesions	[41]
miR-143	It has emerged as potential diagnostic biomarker	Ununified screening protocols for miR-143 hinder the verification of its actual diagnostic value	[50]

miRNA: microRNA

### Protein biomarkers

#### Proteins detected in urine

NMP22 is a biomarker used in the diagnosis of BCa, particularly useful in detecting disease recurrence and in assessing symptoms suggestive of cancer. Its tests are available in quantitative and qualitative forms, each of which can be performed in the laboratory or as a bedside test, which makes it very convenient to use and highly popular. Studies show that the quantitative NMP22 test achieves a sensitivity of 69% and a specificity of 77%. NMP22 sensitivity increases with more advanced stages and higher malignancy of the tumor, but its effectiveness is limited to tumors of low aggressiveness [51]. Bladder tumor antigen (BTA) is a protein biomarker detected in urine, used in the diagnosis of BCa. Its diagnostics include two variants of the test: BTA Stat—a rapid, immunochromatographic spot test that gives results within five minutes; and BTA TRAK—a more detailed, quantitative ELISA test. Both tests detect a protein structurally similar to complement factor H, which BCa cells secrete in excess. BTA Stat demonstrates specificity and sensitivity of 64–93% and 56–83%, respectively, and BTA TRAK of 51–98%, depending on the stage of cancer advancement [52–54]. CYFRA 21.1 is a protein biomarker detected in urine that plays an important role in the diagnosis of BCa, especially when distinguishing local from metastatic disease. This test, based on the ELISA method, enables detection of soluble fragments of cytokeratin 19. It is characterized by high specificity (73–86%) and sensitivity (70–90%). CYFRA 21.1 effectively identifies high-grade tumors and CIS, but false positive results may occur in patients with infections, bladder stones or after intravesical infusions. The test is also useful in monitoring response to chemotherapy [54]. Tumor-associated trypsin inhibitor (TATI) is a peptide detected in many healthy tissues, mainly the gastrointestinal and genitourinary tracts, but its urinary levels increase significantly in various tumors, such as gynecological, lung, breast and bladder. Increased TATI levels are also observed in renal failure, as this peptide is eliminated by renal excretion. In cancers, TATI levels are correlated with tumor presence and aggressiveness, co-expression with tumor-associated trypsin, and acute phase reaction due to tumor invasion. In BCa, TATI levels may vary with stage: higher levels are more characteristic of noninvasive tumors, while TATI expression decreases in more advanced stages. TATI shows high sensitivity in detecting Ta and T1 tumors, is more specific than NMP22, and may act as an independent prognostic factor [53].

Fibrinogen degradation products (FDP) are key biomarkers in BCa, as their levels in urine correlate with the stage of cancer progression. FDP is a breakdown product of fibrin, a protein that accumulates in cancer cells and promotes their growth and metastasis. In patients with BCa, its concentration in urine is significantly elevated, especially in the later stages of the disease, making FDP a valuable marker for monitoring cancer progression. Values ​​above 30 μg/mL often indicate more malignant forms of cancer. FDP also helps distinguish BCa from other conditions, such as urinary tract infections or glomerulonephritis, where its levels may also be elevated, although to a lesser extent. Its role in BCa diagnostics allows earlier detection and more precise monitoring of disease progression, which may lead to better treatment of patients [54, 55]. Interleukin 8 (IL-8) plays an important role in the tumor microenvironment as a mediator of inflammation and angiogenesis, which contributes to the initiation, growth, progression of cancer and metastasis. IL-8 is mainly produced by macrophages and its function is to attract neutrophils, basophils and T cells [56]. The increase in urinary IL-8 concentration, from zero in healthy individuals to 128.43 pg/mL in patients with BCa, indicates its association with carcinogenesis. Elevated IL-8 levels also correlate with disease progression and tumor recurrence, making it a promising prognostic biomarker [57, 58]. COX-2 is a key proinflammatory enzyme, the expression of which is increased in many tumors. In BCa, COX-2 promotes carcinogenesis, resistance to therapy, proliferation, angiogenesis and metastasis. It is produced in the tumor microenvironment by fibroblasts, macrophages, and cancer cells, and its overexpression correlates with poorer prognosis. Although high COX-2 levels are a prognostic marker for BCa recurrence and progression, meta-analyses have shown that its expression is not always directly associated with patient survival, especially in noninvasive tumors [59].

### Metabolomic biomarkers

In a study conducted in 2022, ultra-performance liquid chromatography-mass spectrometry (UPLC-MS) was used to profile metabolites in urine samples collected from 29 patients with BCa and 15 healthy individuals. By analyzing the differences in metabolite expression between groups, a total of 19 differential biomarkers were identified, which were mainly related to metabolic pathways such as fatty acid metabolism, phenylacetate, propanoate, pyruvate, as well as amino acid metabolism such as arginine, proline, glycine, and serine, and bile acid biosynthesis. Of these nineteen metabolites, eleven were selected as key potential diagnostic biomarkers for BCa. These included: glycochenodeoxycholic acid (GCDCA), adenosine monophosphate (AMP), 5-aminolevulinic acid, myristic acid, chenodeoxycholic acid (CDCA), salicyluric acid, proline, *N*-acetylserine, picolinic acid, hydroxypropionic acid and 4-hydroxybenzoic acid. In order to assess their diagnostic usefulness, an analysis was performed using a logistic regression model, which showed excellent diagnostic power. The results obtained from the ROC curve showed that the model achieved an AUC value of 0.983, with a sensitivity of 95.3% and a specificity of 100%. These indicators confirm the high effectiveness of these biomarkers in distinguishing patients with BCa from healthy individuals [60]. According to a 2016 article, BCa affects many metabolic pathways that are key to generating energy, such as glycolysis, the tricarboxylic cycle (TCA), β-oxidation of fatty acids, carnitine metabolism, and amino acid metabolism. In BCa, tryptophan metabolism is increased, which is related to the need for cancer cells to produce more energy to support their rapid growth and proliferation. Additionally, glutathione (GSH) levels increase in response to increased oxidative stress, which is typical for cancer cells that need to counteract oxidative damage. Another important aspect is the increased metabolism of purines and pyrimidines, which contributes to the intensive synthesis of nucleic acids necessary for rapid tumor growth. Disturbances in the levels of choline metabolites have been observed in the urine, serum, and tissues of patients with BCa, suggesting their excessive use for the formation of new cell membranes. Also of interest are the changes in the levels of some metabolites that may distinguish early from advanced BCa stages, such as glucose, lactate, tyrosine, phenylalanine, glycine, and hypoxanthine and uric acid. These results suggest that advanced BCa stages require more intensified metabolism, which is reflected in specific metabolic profiles for each stage [61]. Studies by Sahu et al. [62] have shown that metabolomics can identify metabolic differences between BCa patients and healthy individuals. They identified metabolic signatures including glucose metabolism, the TCA cycle, lipids, amino acids, and nucleotides using GC-MS and LC-MS. Clinical studies conducted on urine samples have shown differences in metabolites such as hippurate, citrate, and taurine, which may be indicators of BCa. In clinical validation, the results showed that the precision values ​​were in line with the Food and Drug Administration (FDA) guidelines, confirming the usefulness of these biomarkers in the diagnosis of BCa [62]. It is also worth mentioning that Jin et al. [63] identified markers related to glycolysis and β-oxidation using LC-MS, and multivariate regression analysis confirmed the correlation between metabolic profiles and patient survival. Integration of such approaches may significantly contribute to improving the diagnosis and treatment of BCa [63].

## Biomarkers in BCa diagnosis

**Figure 3:**
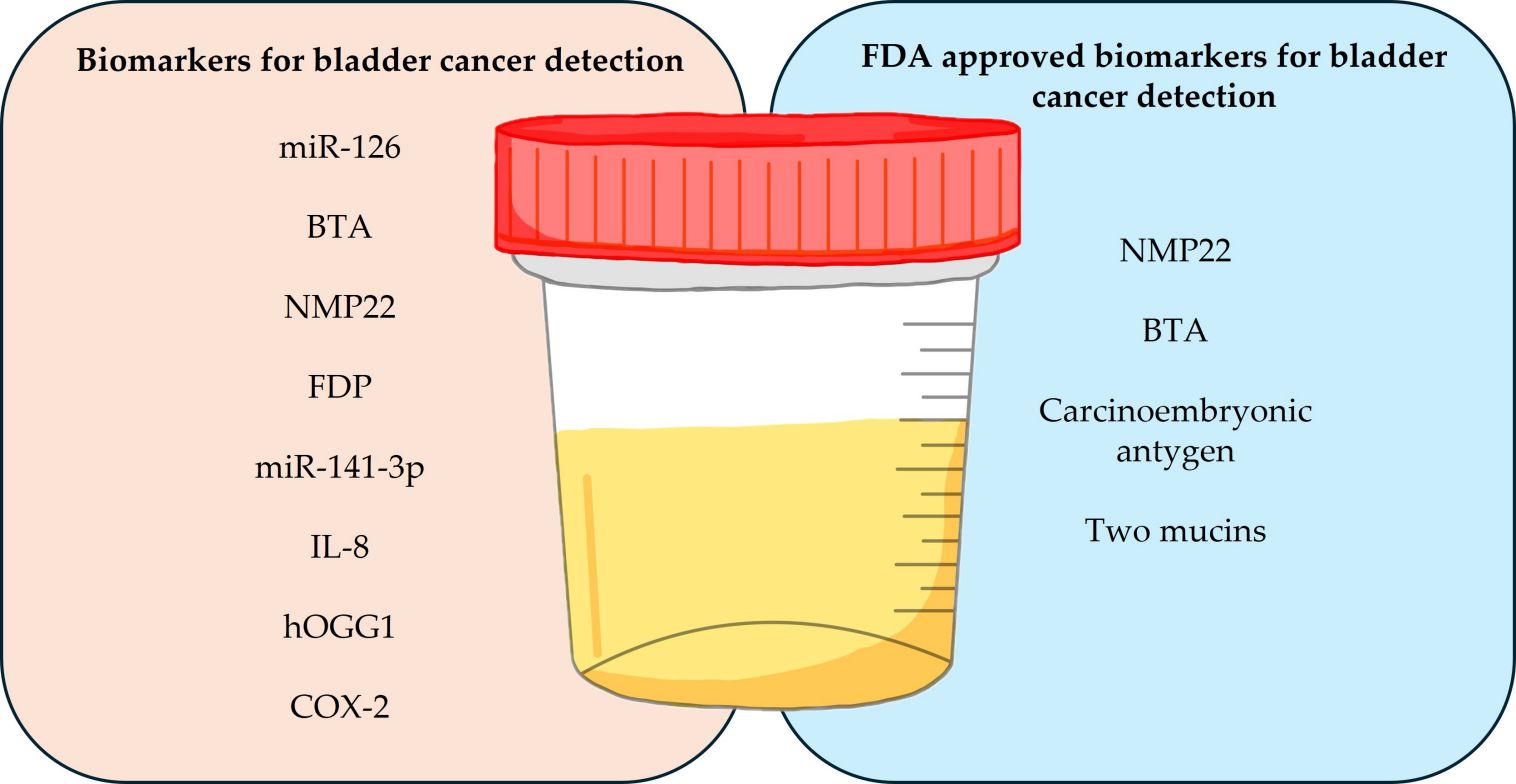
**Summary of biomarkers used in the diagnosis of bladder cancer in urine samples**. miR: microRNA; BTA: bladder tumor antigen; NMP22: nuclear matrix protein 22; FDP: fibrinogen degradation products; IL-8: interleukin 8; FDA: Food and Drug Administration

Below is Table 3, which provides an overview of selected diagnostic tests used in BCa monitoring based on urine analysis. It includes information on the technologies used in these tests, the types of biomarkers that are tested, and a brief description of their performance. Each test has unique diagnostic features, such as sensitivity, specificity, and clinical utility, which allows for a comparison of their performance in detecting BCa, both at the primary stage and in monitoring recurrent disease. Biomarkers used in the diagnosis of BCa, such as miR-126, NMP22, BTA, and hOGG1, as well as those approved by the FDA, are summarized in Figure 3.

**Table 3:** Selected diagnostic tests for BCa

**Name**	**Material**	**Technology**	**Biomarker**	**Pros and cons**	**References**
Cytology	Urine	Giemsa and H&E staining	Cell phenotype	Urine cytology, used in conjunction with cystoscopy, is the classic noninvasive diagnostic test for BCa. It is easy to perform, inexpensive, and can achieve a specificity of up to 98% in detecting high-grade tumors. However, its sensitivity (SN) is low (less than 40%), which limits its usefulness as a stand-alone diagnostic tool.	[64]
NMP22	Urine	ELISA	Peptide	In the case of NMP22 detection tests in patients with microscopic hematuria (three or more red blood cells in the field of view), the NMP22 test showed higher SN (70%) compared with cytology alone (27%). The SN of this test depends on the stage of the tumor, being lower in detecting low-grade lesions. The presence of NMP22 in normal urothelial cells can lead to false positive results, which reduces the specificity of the test to 80%.	[65]
UroVysion	Urine	RYBA	DNA	UroVysion is a test that identifies common chromosomal abnormalities associated with BCa, including changes in chromosomes 3, 7, and 17, and the most common deletion 9p21. The test is expensive and requires specialized equipment and trained personnel. A comparative study showed that UroVysion had higher SN (62% compared to urine cytology) but lower specificity. It is recommended that UroVysion be used only in high-risk patients, especially in cases where urine cytology is equivocal.	[66, 67]
BTA	Urine	Immuno-analysis using a test strip	Protein	The BTA test, approved for monitoring patients with NMIBCa, is rapid and does not require special urine preparation, making it a convenient tool. Studies have shown that BTA Stat has a higher SN than cytology (56%), although a lower specificity (85.7%). In a group of 194 patients with BCa and 185 controls, the SN increased to 73.6%, and the specificity was 83.3%. However, this test tends to produce false positive results in cases of inflammation and previous BCG instillations, which reduces its specificity.	[68, 69]
UroSEEK	Urine	Massively parallel sequencing study	DNA	The UroSEEK test, based on next-generation sequencing (NGS) technology, detects ten mutations typical of BCa, such as FGFR3, TP53, ERBB2, CDKN2A and alterations in the TERTp region by Sanger sequencing. Its main role is to complement cytological diagnostics, rather than replace it. It shows higher SN than cytology, achieving better results in the monitoring (71%) and primary detection (95%) groups. It is also worth noting that the test has lower SN in detecting minimal amounts of mutations, which may limit its accuracy in such cases.	[70]
EpiCheck	Urine	Real-time PCR	DNA	EpiCheck, the only test on the market, uses 15 urinary DNA methylation markers to detect BCa recurrence. The EpiScore algorithm rates the probability of cancer on a scale of 0 to 100. In a study of 353 patients with NMIBCa, EpiCheck showed a SN of 68.2%, specificity of 88.0%, and negative predictive value (NPV) of 95.1%. After excluding low-grade recurrences, its AUC was 0.94, suggesting that it is effective in monitoring high-risk patients. EpiCheck is superior to cytology in SN (62.3%), although it is less specific (86.3%). Additionally, inflammation does not affect the results, which is undoubtedly a great advantage, although the test is expensive and requires specialist handling.	[71, 72]
CxBladder	Urine	RT-qPCR	RNA	CxBladder is a test that uses five RNA markers (CDC2, HOXA13, MDK, IGFBP5, CXCR2) and analyzes their expression using PCR. The algorithms (CxBladder-D and CxBladder-S) assess the risk of cancer and its recurrence. In studies, it has shown greater SN (over 80%) than cytology and NMP22 tests, with a specificity of 85%. CxBladder can also distinguish between low- and high-risk cancers, allowing for better management of patient priorities in the diagnosis and monitoring of BCa.	[73]
Xpert Detection	Urine	RT-qPCR	RNA	Xpert Bladder Cancer Detection is a rapid and noninvasive urine test that detects BCa by analyzing the expression of five mRNA markers (ABL1, ANXA10, UPK1B, CRH, IGF2) using the automated GeneXpert system and RT-PCR technology. The test has a high SN of 78%, especially in high-grade tumors, where SN is 90%, and a high NPV of 98%. Compared to cytology and UroVysion, Xpert offers better results in terms of detecting BCa, making it an effective tool to exclude the disease in patients with hematuria and low risk of BCa.	[74]
Uromonitor	Urine	Real-time PCR	DNA	Uromonitor is a test for detecting mutations in the *TERTp* and *FGFR3* genes in the DNA of cancer cells present in urine, based on real-time PCR technology. The urine filtration system allows for pre-processing and preservation of the sample. In the validation study, Uromonitor achieved 73.5% SN and 73.2% specificity in detecting BCa recurrence, which was similar to the results of cystoscopy. Uromonitor-V2, which adds detection of mutations in the *KRAS* gene, achieved 100% SN and 83.3% specificity in the group of patients with NMIBCa, making it an effective tool for monitoring BCa.	[33, 75]

BCa: bladder cancer; BTA: bladder tumor antigen; FGFR3: fibroblast growth factor receptor 3; NMP22: nuclear matrix protein 22; MIBCa: muscle-invasive BCa; NMIBCa: non-muscle invasive BCa

After conducting a detailed analysis of the Google Patents database, publications regarding the latest patent applications related to the use of biomarkers in the diagnosis of BCa were identified. The summary of these studies is presented in Table 4.

**Table 4:** The summary of most recent studies

**Number**	**Conducted research**	**References**
1	The analysis of DNA methylation in biological samples of patients. The combination of studied genes includes, among others, *AKAP7*, *DFNA5*, *HSD17B2*, *MTRF1L*, *PLCG*, *RPS6KA1*, *STIM2*, *SULT1C2*, *TRABD*, *TRMT1*, *UST*, and *ZNRD1*.	[76, 77]
2	The analysis of transcriptional biomarkers that can predict patient response to immune-activating therapies, such as TLR9 inhibitors, including vidutolimod.	[78]
3	The development of diagnostic kits and devices for detecting urothelial tumors by assessing the methylation levels of these markers TRPS1, HAND2 and ZNF154.	[79]
4	The use of AI models such as fuzzy logic and deep neural networks to analyze medical images and predict treatment response.	[80]

## Biomarkers monitoring disease relapse

Biomarkers such as circulating tumor DNA (ctDNA) are becoming increasingly promising tools for monitoring BCa recurrence and response to treatment. In a study by Birkenkamp-Demtröder et al. [81], plasma ctDNA levels were significantly higher in patients with metastatic recurrence than in disease-free patients. Interestingly, ctDNA analysis was able to detect recurrences more than 100 days earlier than traditional imaging methods, which opens the possibility of earlier therapeutic intervention. In addition, plasma ctDNA levels changed during treatment, allowing monitoring of the effectiveness of chemotherapy or other therapies. This phenomenon may be particularly useful in the context of minimally invasive monitoring of patients, especially those who have undergone cystectomy, who are at high risk of recurrence. Additionally, urinary ctDNA analysis may complement plasma analysis, although further studies are needed. In this way, ctDNA represents a promising tool for personalized care for patients with advanced BCa, allowing for dynamic adjustment of therapy based on early signs of disease recurrence [81].

Another example described in a 2017 article is the Cxbladder Monitor Test, one example of a biomarker that offers high sensitivity (0.93) and high negative predictive value (0.97) in monitoring BCa recurrence. This test analyzes the expression of five genes (*IGFBP5*, *HOXA13*, *MDK*, *CDK1*, *CXCR2*) in urine samples and takes into account the patient’s clinical data, which allows for reliable “ruling out” of disease recurrence, especially in low-risk patients. This method allows for reducing the number of invasive diagnostic tests, which is particularly important in patients with low-grade malignancies [82].

In a published study on monitoring BCa recurrence, a multi-panel urinary biomarker test was shown to be highly effective in detecting BCa recurrence. The panel consisting of 10 biomarkers (IL-8, MMP9, MMP10, SERPINA1, VEGFA, ANG, CA9, APOE, SERPINE1, and SDC1) showed a combined area under the ROC curve (AUROC) of 0.904, which exceeded the results obtained by other tests such as UroVysion or urine cytology. This test achieved a sensitivity of 79% and a specificity of 88%, which means that it is more effective in detecting recurrent cancer compared to classical methods. Moreover, the biomarker panel significantly better detected low-grade malignancies, which is especially important because classical methods such as urine cytology have low sensitivity in detecting this type of changes. This multi-panel biomarker test offers a promising solution for monitoring patients with a history of BCa, reducing the need for invasive tests such as cystoscopy in patients at lower risk of recurrence [83]. URO17 is a promising biomarker for the diagnosis and monitoring of BCa recurrence. It detects keratin 17 (K17), a protein that is strongly associated with tumor cells and their proliferation. URO17 has been used both for the detection of primary tumors and for monitoring disease recurrence, particularly in patients reporting symptoms such as hematuria, which is a common early symptom of BCa [84, 85]. In retrospective studies, URO17 has shown high sensitivity (up to 100%) and specificity in detecting low- and high-grade tumors. Importantly, studies have shown that URO17 is able to detect BCa recurrence in patients both after treatment and during regular surveillance [86, 87]. Due to these features, URO17 has the potential to be widely implemented as a tool for monitoring patients after BCa treatment, allowing for more precise detection of recurrences and minimizing the need for invasive cystoscopies [88]. However, further prospective studies are needed to confirm its clinical utility in a broad patient population [89, 90].

## Challenges and limitations

### Cost and availability of biomarker tests

Urinary biomarkers, although still in the development phase, may help reduce the number of unnecessary procedures, especially in patients with low-grade tumors. In these patients, the use of biomarkers could reduce the frequency of cystoscopy, both saving money and reducing the risk of complications [91]. For patients with more advanced tumors, biomarkers are a valuable adjunct to cystoscopy, helping to more precisely assess tumor aggressiveness and monitor the effects of therapy. Although current biomarkers have limited specificity, further research and development could significantly increase their clinical utility, leading to reduced health care costs and improved patient outcomes [92, 93]. According to Svatek et al. [94], many studies have focused on the use of urine-based tumor markers for the diagnosis and monitoring of BCa. The lower specificity of these markers often leads to false positive results, which questions their real clinical value. Well-designed studies that could clearly demonstrate their clinical advantage are lacking to date. Potential cost savings could result from the use of markers in surveillance regimens, which could reduce the number of cystoscopies or biopsies in patients with atypical cytology results or suspicious cystoscopy results. However, for urine-based biomarkers to become a viable alternative, they must be more effective than cystoscopy, which will require further refinement [94].

## Latest trends in biomarkers

In a comprehensive review in 2024, valuable information was provided on the development and characteristics of BCa biomarkers using bibliometric and bioinformatic analysis. This revealed the biological functions of these biomarkers and their cellular origin. It highlighted the growing interest in somatic mutations and DNA methylation markers that can serve as early diagnostic indicators in BCa. The study emphasizes that BCa is characterized by high heterogeneity, which may make the use of a single biomarker insufficient. Combining several biomarkers can effectively improve diagnostic precision. It also identified an association between BCa and microorganisms such as *Schistosoma*, and differences in the microbiome between BCa patients and healthy individuals. Biomarkers derived from the immune system may not be optimal for BCa diagnostics, but may predict the response to immunotherapy. The study indicates that direct analysis of biological fluids, such as urine, may be more effective in identifying biomarkers than relying solely on tissue data. The authors also note that certain classes of biomarkers, such as microsatellite instability or metabolites, could be included in future studies, although they pose an analytical challenge [95].

After conducting a detailed analysis of the Google Patents database, publications regarding the latest patent applications related to the use of biomarkers in the diagnosis of BCa were identified.

## Conclusion

This paper reviews biomarkers of BCa that may play a key role in improving the diagnosis, monitoring and treatment of this cancer. BCa remains a significant clinical challenge, and traditional diagnostic methods such as cystoscopy and urine cytology have their limitations related to invasiveness, cost and variable sensitivity. Therefore, increasing attention is being paid to biomarkers that offer the possibility of a less invasive and more precise approach. The molecular, genetic, epigenetic, protein and metabolomic biomarkers presented in this paper, as well as modern tests based on RNA and DNA analysis, show significant potential for improving early detection of BCa, prognosticating its course and assessing the response to therapy. Moreover, the development of non-invasive tests based on urine and plasma analysis is a promising alternative to invasive monitoring methods, especially in the context of monitoring cancer recurrence. Despite numerous promising results from preclinical and clinical studies, there is still a need for further validation of biomarkers to ensure their effectiveness and reliability in clinical practice. In particular, it is worth considering the development of new diagnostic tests based on multi-panel biomarker analyses. Combining different types of markers, such as miRNA, ctDNA, and epigenetic changes, could significantly increase sensitivity and specificity, enabling more comprehensive monitoring of the patient's condition. Such an integrated approach could facilitate early detection of the disease, which is crucial for improving treatment outcomes. Another important area is the development of new biosensors with higher sensitivity, which could detect minimal residual disease (MRD) by analyzing ctDNA traces in plasma or urine. The introduction of advanced materials, such as metal nanoparticles or quantum dots, could significantly improve detection limits, which would translate into faster and more accurate diagnoses and more effective monitoring of disease recurrence. It is also worth considering personalizing diagnostic tests by developing detection platforms based on specific genetic mutations or molecular profiles characteristic of individual patients. Personalizing tests could not only improve treatment efficacy, but also minimize the risk of therapeutic failures by adapting the therapy to the individual needs of the patient. In summary, the discovery and development of biomarkers for BCa is a promising but still developing area of ​​research that requires further investigation. The implementation of more precise, noninvasive diagnostic and monitoring methods, such as integrated biomarker panels, more sensitive biosensors, and personalized tests, could revolutionize the diagnosis and treatment of BCa. Such innovations could contribute not only to improving the quality of care for patients, but also to reducing costs and minimizing invasive procedures in everyday clinical practice.

## Abbreviations

BCa: bladder cancer
BTA: bladder tumor antigen
ctDNA: circulating tumor DNA
FDA: Food and Drug Administration
FDP: fibrinogen degradation products
FGFR3: fibroblast growth factor receptor 3
IL-8: interleukin 8
MIBCa: muscle-invasive bladder cancer
miRNA: microRNA
NLUTD: neurogenic lower urinary tract dysfunction
NMIBCa: non-muscle invasive bladder cancer
NMP22: nuclear matrix protein 22
TATI: tumor-associated trypsin inhibitor

## Declarations

### Availability of data and materials

The raw data supporting the conclusions of this manuscript will be made available by the authors, without undue reservation, to any qualified researcher.

### Copyright

© The Author(s) 2025.
